# The effect of saponins from *Ampelozizyphus amazonicus* Ducke on the renal Na^+^ pumps’ activities and urinary excretion of natriuretic peptides

**DOI:** 10.1186/1472-6882-12-40

**Published:** 2012-04-11

**Authors:** Lúcio Ricardo Leite Diniz, Viviane Gomes Portella, Flávia Magalhães Cardoso, Aloa Machado de Souza, Celso Caruso-Neves, Geovanni Dantas Cassali, Adelina Martha dos Reis, MariadasGraçasLins Brandão, Maria Aparecida Ribeiro Vieira

**Affiliations:** 1 Departamento de Fisiologia e Biofísica, Instituto de Ciências Biológicas, Universidade Federal de Minas Gerais, Av. Antônio Carlos, 6627, Belo Horizonte, Minas Gerais, 31270-901, Brazil; 2 Instituto de Biofísica Carlos Chagas Filho, Universidade Federal do Rio de Janeiro, CCS-Bloco G, Rio de Janeiro, 21949-900, Brazil; 3 Departamento de Patologia Geral, Instituto de Ciências Biológicas, Universidade Federal de Minas Gerais, Av. Antônio Carlos, 6627, Belo Horizonte, Minas Gerais, 31270-901, Brazil; 4 Laboratório de Pharmacognosia, Faculdade de Farmácia, Universidade Federal de Minas Gerais, Av. Antônio Carlos, 6627, Belo Horizonte, Minas Gerais, 31270-901, Brazil

**Keywords:** *Ampelozizyphus amazonicus* Ducke, Rhamnaceae, saponins, antidiuresis, Na^+^-ATPase, (Na^+^,K^+^)-ATPase, atrial natriuretic peptides

## Abstract

**Background:**

In a previous study, we showed that a saponin mixture isolated from the roots of *Ampelozizyphus amazonicus* Ducke (SAP*Aa*D) reduces urine excretion in rats that were given an oral loading of 0.9 % NaCl (4 ml/100 g body weight). In the present study, we investigated whether atrial natriuretic peptides (ANP) and renal ATPases play a role in the SAP*Aa*D- induced antidiuresis in rats.

**Methods:**

To evaluate the effect of SAP*Aa*D on furosemide-induced diuresis, Wistar rats (250-300 g) were given an oral loading of physiological solution (0.9 % NaCl, 4 ml/100 g body weight) to impose a uniform water and salt state. The solution containing furosemide (Furo, 13 mg/kg) was given 30 min after rats were orally treated with 50 mg/kg SAP*Aa*D (*SAPAaD + Furo*) or 0.5 ml of 0.9 % NaCl (*NaCl + Furo*). In the *SAPAaD + NaCl* group, rats were pretreated with SAP*Aa*D and 30 min later they received the oral loading of physiological solution. Animals were individually housed in metabolic cages, and urine volume was measured every 30 min throughout the experiment (3 h). To investigate the role of ANP and renal Na^+^ pumps on antidiuretic effects promoted by SAP*Aa*D, rats were given the physiological solution (as above) containing SAP*Aa*D (50 mg/kg). After 90 min, samples of urine and blood from the last 30 min were collected. Kidneys and atria were also removed after previous anesthesia. ANP was measured by radioimmunoassay (RIA) and renal cortical activities of Na^+^- and (Na^+^,K^+^)-ATPases were calculated from the difference between the [^32^P] Pi released in the absence and presence of 1 mM furosemide/2 mM ouabain and in the absence and presence of 1 mM ouabain, respectively.

**Results:**

It was observed that SAP*Aa*D inhibited furosemide-induced diuresis (at 90 min: from 10.0 ± 1.0 mL, *NaCl + Furo* group, n = 5, to 5.9 ± 1.0 mL, *SAPAaD + Furo* group n = 5, p < 0.05), increased both Na^+^-ATPase (from 25.0 ± 5.9 nmol Pi.mg^-1^.min^-1^, control, to 52.7 ± 8.9 nmol Pi.mg^-1^.min^-1^, p < 0.05) and (Na^+^,K^+^)-ATPase (from 47.8 ± 13.3 nmol Pi.mg^-1^.min^-1^, control, to 79.8 ± 6.9 nmol Pi .mg^-1^.min^-1^, p < 0.05) activities in the renal cortex. SAP*Aa*D also lowered urine ANP (from 792 ± 132 pg/mL, control, to 299 ± 88 pg/mL, p < 0.01) and had no effect on plasma or atrial ANP.

**Conclusion:**

We concluded that the SAP*Aa*D antidiuretic effect may be due to an increase in the renal activities of Na^+^- and (Na^+^,K^+^)-ATPases and/or a decrease in the renal ANP.

## Background

Several studies have reported that some medicinal herbs alter diuresis [1-5] even though little is known about the mechanism(s) that account for this claimed physiological effect. *Ampelozizyphus amazonicus* (*A. amazonicus*) is a Rhamnaceae known as ‘saracura-mirá’ or ‘Indian beer’ that is traditionally used by Brazilian Indians and Cabocos (from the Tupi *kaa'boc*) who live in all regions along Rio Negro, Amazônia. Beverages prepared with their roots are used as stimulant against tiredness, fatigue and starvation [6]). The beverage is also used against malaria, liver disturbances and sleeplessness, and serve as depurative [7].

In a previous studies, we have shown that a triterpene saponin mixture isolated from the roots of *A. amazonicus* Ducke (SAP*Aa*D), reduced the urine excretion of normal rats in a dose-dependent manner [8]. There is a need for novel oral antidiuretics to treat diseases, such as pituitary diabetes insipidus and nephrogenic diabetes insipidus; however, investigations for compounds other than the typically used arginine vasopressin (AVP) or desmopressin are lacking. It is well known that diuresis is regulated by a number of endogenous and exogenous compounds that act at multiple intrarenal sites to influence urine formation [9]. The final urine volume and composition are determined primarily in the renal tubule system without a direct extensive dependence on the glomerular filtration rate. In the renal tubules, water reabsorption is usually secondary to transcellular sodium reabsorption. Additionally, AVP regulates water balance and osmolality by manipulating water permeability that is not obscured by responses to sodium reabsorption [10]. Renal tubular sodium reabsorption involves two primary active transporters: the ouabain-sensitive (Na^+^,K^+^)-ATPase and the furosemide-sensitive Na^+^-ATPase [9-11]. The activity of both ATPases is directly regulated by hormones, such as natriuretic peptides. These hormones play an important role in hydroelectrolytic homeostasis by stimulating natriuresis through the coordination of apical sodium channels and basolateral (Na^+^,K^+^)-ATPases in the inner medullary collecting ducts [12-16]. Over the last few decades, several studies have demonstrated the potential role of the cortical sodium pumps in the composition of excreted urine. The various hormones that play a role in the modulation of urine composition act on these sodium pumps in the cortical segment, particularly in the proximal tubule [17-19]. Accordingly, it is well known that specific modifications of sodium reabsorption in the proximal tubule cells lead to an increase in renal sodium and water excretion as observed in primary hypertension [20-23]. Then, it is plausible to postulate that changes in the cortical sodium pumps promoted by a compound could lead to changes in urine composition. It has been reported that sodium pumps’ activities can be modulated by herbal products, such as *Petroselinum hortense* extracts*,* which inhibit (Na^+^,K^+^)-ATPase, and saponins isolated from *Costus spicatus* Swartz, which inhibit Na^+^-ATPase activity [24,25]. Because the inhibition of renal Na^+^ transport may account for the diuretic effect of different agents, we investigated the renal cortical Na^+^ pumps and renal natriuretic peptides in SAP*Aa*D-induced antidiuresis.

## Methods

### Plant material

The roots of *A. amazonicus* Ducke were collected (September, 2000) in the city of Presidente Figueiredo, Amazonas State, Brazil, and were identified by Dr. Ari Hidalgo. Voucher specimens (189,858) were deposited in the herbarium of the Instituto Nacional de Pesquisa da Amazônia (Manaus/AM, Brazil).

### Extraction, isolation and characterization of SAP*Aa*D

The extraction, isolation and chemical characterization of SAP*Aa*D were performed according to previously described procedures [8]. Powdered roots of *A. amazonicus* were successively extracted by percolation with 70 % ethanol, with the solvent then evaporated to dryness (6.6 % w/w). The crude extract was resuspended in water and treated with n-butanol. After evaporation at a maximum of 60°C, the organic and aqueous phases furnished the SAP*Aa*D and saponin-free (SAP*Aa*D-free) fractions, respectively. Chemical characterisation was performed by HPLC/DAD. Briefly, HPLC analysis was carried out on an Agilent 1200 system (Palo Alto, CA, USA). Column, lichrospher reversed-phase C_18_ (250 mm x 4.6 mm I.D., 5 μm particle size, Merck); mobile phase, acetonitrile (solvent A) and water (solvent B); elution gradient, 30–40 % A at 0–10 min, 40–90 % A at 10–40 min, 90–100 % A at 40–45 min, and 100 % A at 45–50 min; flow rate, 1.0 mL/min; injection volume, 20 μL. For the analysis, 30 mg of SAP*Aa*D were dissolved in 10 mL of acetonitrile:water (30:70) in an ultrasonic bath for 10 min. All solutions were filtered through a 0.45 μm membrane filter before injection. UV photodiode array detection was performed at 205 nm, and UV spectra from 200 to 400 nm were on-line recorded for peak identification.

### Animals

Male Wistar rats weighing 250–300 g were housed in standard conditions with free access to commercial chow and water prior to the experiment. Animals were kept at a room temperature of 22°C with a light/dark cycle of 14/10 h. All procedures described here had prior approval from the Institutional Animal Use Ethics Committee (protocol 177, November, 2008).

### Interaction between furosemide and SAP*Aa*D

To evaluate a possible interaction between furosemide (Lasix®, Aventis Pharma Ltda, São Paulo, Brazil) and SAP*AaD*, all animals were given an oral loading of physiological solution (0.9 % NaCl, 4 ml/100 g body weight) to impose a uniform water and salt state. The physiological solution containing furosemide (13 mg/kg) was given 30 min after rats were orally treated with 50 mg/kg SAP*Aa*D (*SAPAaD + Furo* group) or 0.5 ml of 0.9 % NaCl (*NaCl + Furo* group). Rats of group *SAPAaD + NaCl* were pretreated with SAP*Aa*D and 30 min later they received the oral loading of physiological solution. Rats in the *NaCl + AVP* group received the physiological solution containing 200 ng/kg AVP (Sigma Chemical Co., St Louis, MO, USA, 98 % purity) after (30 min) rats were orally treated with 0.5 ml of 0.9 % NaCl. Rats in the *NaCl + NaCl* group received the oral loading of physiological solution after pretreatment with 0.5 ml of 0.9 % NaCl (control). Animals were individually housed in metabolic cages, and urine volume was measured every 30 min throughout the experiment (3 h).

In a series of experiments, rats were given the oral loading of physiological solution as above in the absence (control) and presence of 50 mg/kg SAP*Aa*D. At 90 min after SAP*Aa*D administration, the urine volume was measured, and a sample of urine was collected and stored at −20°C until the measurement of urine ANP was performed. Afterward, the rats were anesthetized with 40 mg/kg of thiopental, and a blood sample was drawn from the inferior cava vein. Under anesthesia, the rats were sacrificed, and the kidneys were removed for the histomorphological analysis and the renal ATPase activity measurements. Both atria were also removed to determine the ANP content. Blood samples were centrifuged at 3,000 rpm for 10 min at 4°C, and the recovered plasma was stored at −80°C until the ANP assay was performed. For the histomorphological analysis, sections of the kidney were obtained from representative animals in the control and SAP*Aa*D-treated groups. The tissue was fixed in 10 % formalin, embedded in paraffin and was dissected into 4 μm sections. The sections were stained with hematoxylin and eosin and were examined under a light microscope.

### Measurement of renal ATPases’ activities

The kidneys were removed immediately after the rats were sacrificed under anesthesia and were maintained in a cold isosmotic solution containing 250 mM sucrose, 10 mM HEPES-Tris, pH 7.6, 2 mM ethylenediaminetetraacetic acid (EDTA) and 1 mM phenylmethylsulfonyl fluoride (PMSF). Thin slices of cortex (cortex-corticis) were separated using a scalpel and homogenized in the same cold solution with a teflon and glass homogenizer. The homogenate was centrifuged for 10 min at 10,000 rpm in a Sorvall centrifuge using an SS-34 rotor at 4°C. The pellet was discarded and the supernatant was centrifuged for 1 h at 60,000 rpm at 4°C. The membrane pellet containing the microsomal fraction was resuspended in 250 mM sucrose to a final concentration of 6–10 mg of protein per milliliter and was stored at 4°C. ATPase activity was measured as previously reported [26-28]. Na^+^-ATPase and (Na^+^,K^+^)-ATPase activities expressed in nmol Pi x mg^-1^ x min^-1^ were obtained by subtracting the enzyme activity in the absence and presence of furosemide (2 mM) and ouabain (1 mM), respectively [27,28].

### Measurement of atrial natriuretic peptides

To determine the atrial content of ANP, the left and right atria were pooled in a prechilled tube containing a cocktail of protease inhibitors consisting of 0.1 M acetic acid, 10^-5^ mol/L EDTA, 10^-5^ mol/L PMSF and 0.5 x 10^-5^ mol/L pepstatin A (Sigma Chemical Co., St. Louis, MO, USA). The tube contents were homogenized (Euroturrax homogenizer T20b; Janke and Kunkel Ika Labortechnik, Staufen, Germany) and centrifuged at 20,000 x g for 30 min at 4°C. The supernatants were diluted in a phosphosaline buffer (0.01 mol/L sodium phosphate, 0.14 mmol/L bovine serum albumin, 0.10 % Triton X-100, 0.10 mol/L NaCl and 0.01 % sodium azide, pH 7.4), and ANP content was determined by radioimmunoassay (RIA) [29]. ANP antibody was kindly donated by Dr. J Gutkowska (CHUM – Université de Montreal, Montreal, Canada). Standard ANP was obtained from Bachem Inc. (Torrance, CA, USA). The protein concentration in the supernatant was measured as previously described [30]. Atrial ANP was normalized to protein concentration and reported in micrograms per milligram of protein. To quantify plasma ANP, plasma samples (1 mL) were extracted in Sep-Pak C_18_ cartridges (Waters Corporation, Milford, MA, USA). Eluents were evaporated until dry in a Speed-Vac (Eppendorf 5301, Hamburg, Germany), and the resultant powder was dissolved in 500 μL of phosphosaline buffer prior to determining the ANP concentration by RIA [29]. To quantify the urine ANP, 1 mL of urine was extracted using Sep-Pak C_18_ cartridges (Waters Corporation, Milford, MA, USA), as previously described [29]. After being evaporated until dry in a Speed-Vac, the samples were dissolved in 500 μL of phosphosaline buffer, and urine ANP was determined by RIA using anti-ANP antibodies. Because atrial ANP and urine ANP have a very similar structure with an identical C-terminus [31], anti-ANP antibodies may also be employed to measure ANP in urine. In addition, insignificant concentrations of circulating ANP are found in the urine because of high ANP degradation from peptidases in the kidney cortex membranes or because of ANP binding to the clearance C receptor [32,33].

### Statistical analysis

The results were expressed as means ± standard error of the mean (± SEM). The data shown the effect of SAPAaD and furosemide on diuresis were analyzed using nonlinear regression and two-way ANOVA followed by Bonferroni’s test. To analyze the other investigated parameters, unpaired Student’s *t*-test was also employed to compare the control and SAP*Aa*D-treated groups. A p-value less than 0.05 (p < 0.05) was considered statistically significant.

**Figure 1:**
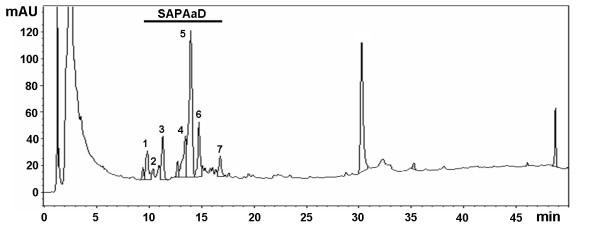
**HPLC fingerprinting of SAP*Aa*D.** The retention times of saponins are (in min): **1**–9.42, **2**–9.85, **3**–11.32, **4**–13.46, **5**–13.79, **6**–14.75 and 7–16.78. Detection, 205 nm; mAU, milli absorbance unit.

## Results

Figure 1 shows a typical fingerprint chromatogram of SAP*Aa*D mixture. The retention times of saponins were between 9.42 and 16.78 min. The purification process performed to obtain saponins was very efficient since the peaks related to saponins were predominant in the SAP*Aa*D fraction fingerprint.

**Figure 2:**
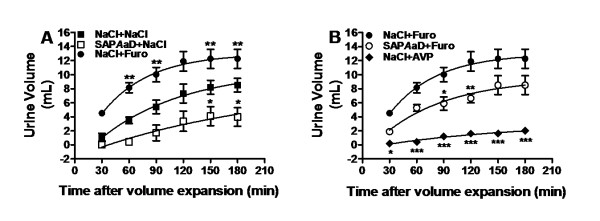
**Effect of SAP*Aa*D and furosemide on diuresis in rats.** Animals were given an oral loading of physiological solution (0.9 % NaCl, 4 ml/100 g body weight) to impose a uniform water and salt state. In **A**: Rats were orally pretreated (30 min) with 0.5 ml 0.9 % NaCl and received the physiological solution alone (*NaCl + NaCl*) or containing 13 mg/kg furosemide (*NaCl + Furo*); *SAPAaD + NaCl,* rats were orally pretreated with 50 mg/kg SAP*Aa*D and received the physiological solution alone. In **B**: Rats were orally pretreated with SAP*Aa*D and received the physiological solution containing furosemide (*SAPAaD + Furo*); *NaCl + AVP*, rats were orally pretreated with 0.5 ml 0.9 % NaCl and received the physiological solution containing AVP. *NaCl + Furo* is the same as in **A**. Animals (n = 5/group) were individually housed in metabolic cages, and urine volume was measured every 30 min throughout the experiment (3 h). Data are expressed as means ± SEM. In **A**, *p < 0.05 vs. group *NaCl + NaCl*. In **B**,*p <0.05, **p < 0.01 and ***p < 0.001 vs. group *NaCl + Furo* at the correspondent time. Data were analyzed by two-way ANOVA followed by Bonferroni’s test.

All experiments using animals were performed in rats with a 4 % body weight volume expansion (oral loading of 0.9 % NaCl, 4 ml/100 g body weight) because this magnitude of volume expansion is an intermediate value between the experimentally obtainable minimum and maximum values used for studies examining the acute effect of substances that either stimulate or inhibit diuresis. In this study, SAP*Aa*D was tested at the dose of 50 mg/kg because it resulted in an approximate 60 % reduction in the diuresis induced by the oral loading of 0.9 % NaCl (4 ml/100 g body weight) in normal rats. This reduction was reported in our previous study [8] in which we have shown that SAP*Aa*D at dose varying from 25 to 1000 mg/kg reduced the urine production in a dose-dependent manner. As 60 % reduction neither represent the minimum nor the maximum effect of SAP*Aa*D, 50 mg/kg was the dose of choice to perform the experiments in the present study. No significant alteration of renal morphology was observed after SAP*Aa*D treatment (data not shown).

### Interaction between furosemide and SAP*Aa*D

As shown in Figure 2, SAP*Aa*D and furosemide have antagonistic effects on diuresis. While SAP*Aa*D reduced the diuresis elicited by the oral loading of 0.9 % NaCl (at 180 min: from 8.5 ± 1.0 mL, *NaCl + NaCl* group, to 4.0 ± 1.3 mL, *SAPAaD + NaCl* group, n = 5) (Figure 2A), furosemide dosed at 13 mg/kg greatly increased urine elimination in rats that equally received oral 0.9 % NaCl (at 180 min: from 8.5 ± 1.0 mL to 12.2 ± 1.4 mL, *NaCl + Furo* group, n = 5) (Figure 2A). However, pretreatment with SAP*Aa*D eliminates this increase in the furosemide-induced diuresis (Figure 2B). In the presence of SAP*Aa*D, the urine volume produced by furosemide (8.5 ± 1.3 mL, *SAPAaD + Furo* group n = 5) (Figure 2B) was similar to that observed in the *NaCl + NaCl* group (8.5 ± 1.0 mL, n = 5) (Figure 2A) at 180 min. It is noteworthy that SAP*Aa*D, although in a less extent, induced antidiuresis as AVP, which was used as a positive control (Figure 2B).

### Effect of SAP*Aa*D on renal ATPases’ activities and atrial natriuretic peptides

**Figure 3:**
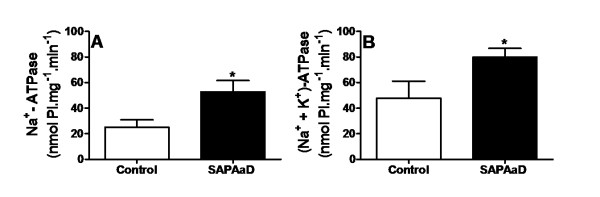
**Effect of SAP*Aa*D on renal Na**^**+**^**- (A) and (Na**^**+**^**, K**^**+**^**)-ATPase activities (B).** Rats were given an oral loading of physiological solution (0.9 % NaCl, 4 ml/100 g body weight) in the absence (control) and presence of 50 mg/kg of SAP*Aa*D). At 90 min after SAP*Aa*D administration, rats were sacrificed, and the kidneys were removed for renal cortical ATPase measurement. ATPases’ activities were expressed as nmol Pi per milligram of total protein per minute. Data are expressed as means ± SEM of four rats/group. *p < 0.05 compared with the control group, unpaired Student’s *t*-test.

**Figure 4:**
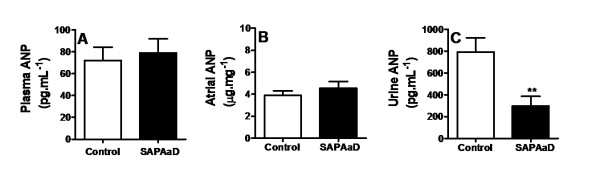
**Effects of SAP*Aa*D on plasma (A), atria (B) and urine (C) ANP.** Rats received an oral loading of physiological solution (0.9 % NaCl, 4 ml/100 g body weight) in the absence (control) and presence of 50 mg/kg of SAP*Aa*D). At 90 min after SAP*Aa*D administration, a sample of blood and urine were collected, rats were sacrificed and the atria were removed for ANP measurement. Plasma (n = 6), pooled left and right atria (n = 6) and urine ANP (n = 7) were measured by RIA. Atria ANP were normalized to protein concentration and reported in micrograms per milligram of protein. Data are represented as means ± SEM. ANP in control: Plasma and atria (n = 12) and urine (n = 7). **p < 0.01 compared with the control group, unpaired Student’s *t*-test.

SAP*Aa*D (50 mg/kg) significantly increased the activities of renal Na^+^-ATPase (from 25.0 ± 5.9 nmol Pi. mg^-1^. min^-1^, control, n = 4, to 52.7 ± 8.9 nmol Pi.mg^-1^. min^-1^, n = 4, p < 0.05) (Figure 3A) and renal (Na^+^, K^+^)-ATPase (from 47.8 ± 13.3 nmol Pi.mg^-1^.min^-1^, control, n = 4, to 79.8 ± 6.9 nmol Pi.mg^-1^.min^-1^, n = 4, p < 0.05) (Figure 3B). The ANP levels in both the plasma (Figure 4A, n = 6) and atria (Figure 4B, n = 6) were not significantly affected by SAP*Aa*D at the 50 mg/kg dosage. In contrast, urine ANP excretion was significantly reduced in the rats treated with SAP*Aa*D (from 792 ± 132 pg/mL, control, n = 7, to 299 ± 88 pg/mL, n = 7, p < 0.01) (Figue 4C).

## Discussion

In this study, we showed for the first time that saponins, in vivo, stimulate renal ATPases and reduce the level of urine ANP. These effects are undoubtedly characteristic of an antidiuretic agent. Moreover, SAP*Aa*D abolished the diuretic effect of furosemide, a classical diuretic that affects renal Na^+^-ATPases [32]. In a previous study, our group reported that the oral administration of SAP*Aa*D exhibited a dose-dependent antidiuretic effect in normal rats in either dehydrated or hydrated conditions [8]. In the present study, SAP*Aa*D exhibited a similar effect even when administered 30 min prior to an oral loading of physiological solution (0.9 % NaCl). Because pretreatment with SAP*Aa*D prevented the effect of furosemide, one could expect that SAP*Aa*D might share the same mechanistic pathway by which furosemide enhances diuresis. According to Becker et al. [34], the diuretic effect of furosemide was reduced in hypertensive patients with domiciliary use of a nutritional supplement derived from ginseng. Ginseng is a natural compound that is rich in triterpene saponins structurally similar to SAP*Aa*D. It is known that furosemide inhibits renal Na^+^-ATPase activity [11,32]. In addition, some studies have reported that the activities of renal ATPases may be modulated by medicinal plants. It has been shown that the diuretic effect of crude *Petroselinum hortense* extract is accompanied by the inhibition of (Na^+^,K^+^)-ATPase activity [25]. Na^+^-ATPase was similarly inhibited by steroidal saponins isolated from *Costus spicatus*, a plant used in Brazilian folk medicine to expel kidney stones [24]. In contrast, the present study shows SAP*Aa*D in vivo produced a significant increase in both Na^+^- and (Na^+^,K^+^)-ATPase activities. These data suggest that SAP*Aa*D decreased the diuresis, at least in part, by stimulating renal ATPases. Accordingly, it was previously reported that saponins unmask a latent intracellular pool of (Na^+^,K^+^)-ATPase leading to an increase in the renal tubular ATPase activity [35,36]. It has been reported that endogenous compounds, such as ANP_99-126_ and urodilatin [12-14], stimulate natriuresis and diuresis primarily by coordinating the inhibition of apical Na^+^ channels and basolateral (Na^+^,K^+^)-ATPases in the inner medullary collecting ducts [12]. However, a possible role of the cortical sodium pumps in the urine final composition cannot be ruled out. Different hormones that modulate the urine composition act on these cortical sodium pumps located particularly in the proximal tubules [17-19]. It is well established that specific modifications in sodium reabsorption in proximal tubule cells lead to an increase in renal sodium and water excretion as observed in primary hypertension [20-23]. These observations indicate that changes in the cortical sodium pumps may lead to changes in urine composition. In addition, alteration in the renal and/or plasma levels of the natriuretic peptides may play a role in the SAP*Aa*D-induced antidiuresis. A significant reduction in the urine ANP concentration was observed in the urine of rats treated with SAP*Aa*D. Therefore, we cannot exclude the possibility that the increase in renal ATPase activity induced by SAP*Aa*D was a result of the reduction in the renal urodilatin level. This explanation is, at least in part, consistent with the observation that renal urodilatin inhibits Na^+^-ATPase activity but not (Na^+^,K^+^)-ATPase activity in proximal tubules [37]. However, in a previous study [38], it was shown that exogenous urodilatin dosed at 10 nM increased the (Na^+^,K^+^)-ATPase activity in the renal outer cortex of Sprague–Dawley rats. The discrepancy between this study and our investigation may be because (Na^+^,K^+^)-ATPase activity was measured in the whole cortex and because the urine ANP excretion was lower than the exogenous urodilatin used in the previous study (10 nM). Natriuretic peptides are very important hormones that regulate renal processes since they are effective at promoting changes in renal vasculature by acting as vasodilators, and renal tubules by reducing Na^+^ and water reabsorption. In our study, the reduction in the urine ANP may denote a decreased intrarenal ANP content. In turn, the low level of renal ANP would not modulate the activity of renal cortical Na^+^ pumps resulting in a higher Na^+^ reabsorption. As a consequence, the urinary elimination of salts and water is diminished.

## Conclusions

Although the precise mechanism by which SAP*Aa*D affect diuresis is not completely elucidated, our data indicate that the antidiuretic effect of SAP*Aa*D may be due to a reduction in the renal ANP levels and/or an increase in the renal ATPase activity. We also conclude that saponins from *A. amazonicus* Ducke might be an herbal candidate for an antidiuretic with therapeutic potential. On the other hand, the *A. amazonicus* Ducke species should be used with caution because antidiuretic agents should be avoided in certain conditions, such as hypertension.

## Abbreviations

ANP: atrial natriuretic peptide; AVP: arginine vasopressin; Furo: furosemide; HPLC/DAD: high performance liquid-chromatography with diode array detectors; SAPAaD: saponin mixture isolated from the roots of Ampelozizyphus amazonicus Ducke.

## Competing interests

The authors declare that they have no competing interests.

## Author´s contributions

LRLD designed and performed all of the experimental protocols, took part in all of the analytical procedures, analysed the data and prepared the manuscript; VGP designed and performed the experimental protocols; FMC performed the RIAs; AMdS performed the renal Na^+^-pumps activities; CCN designed the analytical protocol to measure the renal Na^+^-pumps activities and prepared the manuscript; GDC, performed the histomorphological analysis; AMdR designed the RIAs analysis and analysed the data; MdGLB performed the extraction, isolation and characterization of SAPAaD; MARV designed and supervised the experiments, analysed the data and prepared the manuscript. All authors read and approved the final manuscript.

## Pre-publication history

The pre-publication history for this paper can be accessed here:

http://www.biomedcentral.com/1472-6882/12/40/prepub

## References

[B1] Wright CI Van-Buren L Kroner CI Koning MM Herbal medicines as diuretics: a review of the scientific evidence J Ethnopharmacol 2007 114 1 1 31 10.1016/j.jep.2007.07.023 17804183

[B2] Lahlou S Tahraoui A Israili Z Lyoussi B Diuretic activity of the aqueous extracts ofCarum carviand Tanacetum vulgare in normal rats J Ethnopharmacol 2007 110 3 458 463 10.1016/j.jep.2006.10.005 17113735

[B3] Haloui M Louedec L Michel JB Lyoussi B Experimental diuretic effects ofRosmarinus officinalisandCentaurium erythraea J Ethnopharmacol 2000 71 3 465 472 10.1016/S0378-8741(00)00184-7 10940584

[B4] Tahri A Yamani S Legssyer A Aziz M Mekhfi H Bnouham M Ziyyat A Acute diuretic, natriuretic and hypotensive effects of a continuous perfusion of aqueous extract ofUrtica dioicain the rat J Ethnopharmacol 2000 73 1–2 95 100 11025144 10.1016/s0378-8741(00)00270-1

[B5] Ganapaty S Dash GK Subburaju T Suresh P Diuretic, laxative and toxicity studies ofCocculus hirsutusaerial parts Fitoterapia 2002 73 1 28 31 10.1016/S0367-326X(01)00345-8 11864760

[B6] Souza Santos AM Kahwage CC Coelho-Ferreira MR Smpaio NA Traditional medicines in the Rio Negro valley (Amazonas state, Brazil). Observation on the ethnopharmacology and the use of the plant Saracura-Mirá (Ampelozizyphus amazonicus): Pharmacological activity and/or symbolic efficacy Bol. Mus. Para. Emílio Goeldi, sér. Ciências Humanas, Belém 2005 1 1 137 147

[B7] Coelho-Ferreira MR Les Plantes Médicinales à Manaus: Utilisation et commercialisation. DESU de Biologie Végétale Tropicale. Paris, Université Paris VI,1992, p.60

[B8] Diniz LR Santana PC Ribeiro AP Portella VG Pacheco LF Meyer NB Cesar IC Cosenza GP Brandao MG Vieira MA Effect of triterpene saponins from roots ofAmpelozizyphus amazonicusDucke on diuresis in rats J Ethnopharmacol 2009 123 2 275 279 10.1016/j.jep.2009.03.006 19429372

[B9] Matsubara M Renal sodium handling for body fluid maintenance and blood pressure regulation Yakugaku Zasshi 2004 124 6 301 309 10.1248/yakushi.124.301 15170065

[B10] Stockand JD Vasopressin regulation of renal sodium excretion Kidney Int 2010 78 9 849 856 10.1038/ki.2010.276 20736986

[B11] Proverbio F Proverbio T Marin R Na + −ATPase is a different entity from the (Na++ K+)-ATPase in rat kidney basolateral plasma membranes Biochim Biophys Acta 1986 858 1 202 205 10.1016/0005-2736(86)90307-X 3011092

[B12] Beltowski J Wojcicka G Regulation of renal tubular sodium transport by cardiac natriuretic peptides: two decades of research Med Sci Monit 2002 8 2 RA39 52 11859295

[B13] Ewart HS Klip A Hormonal regulation of the Na(+)-K(+)-ATPase: mechanisms underlying rapid and sustained changes in pump activity Am J Physiol 1995 269 2 Pt 1 C295 311 7653511 10.1152/ajpcell.1995.269.2.C295

[B14] Bestle MH Bie P Renal effects of urodilatin and atrial natriuretic peptide in volume expanded conscious dogs Acta Physiol Scand 1993 149 1 77 83 10.1111/j.1748-1716.1993.tb09594.x 8237425

[B15] Hildebrandt DA Mizelle HL Brands MW Hall JE Comparison of renal actions of urodilatin and atrial natriuretic peptide Am J Physiol 1992 262 3 Pt 2 R395 399 1313650 10.1152/ajpregu.1992.262.3.R395

[B16] Meyer M Richter R Brunkhorst R Wrenger E Schulz-Knappe P Kist A Mentz P Brabant EG Koch KM Rechkemmer G Urodilatin is involved in sodium homeostasis and exerts sodium-state-dependent natriuretic and diuretic effects Am J Physiol 1996 271 3 Pt 2 F489 497 8853410 10.1152/ajprenal.1996.271.3.F489

[B17] Feraille E Doucet A Sodium-potassium-adenosinetriphosphatase-dependent sodium transport in the kidney: hormonal control Physiol Rev 2001 81 1 345 418 11152761 10.1152/physrev.2001.81.1.345

[B18] Lara Lda S Cavalcante F Axelband F De Souza AM Lopes AG Caruso-Neves C Involvement of the Gi/o/cGMP/PKG pathway in the AT2-mediated inhibition of outer cortex proximal tubule Na+−ATPase by Ang-(1–7) Biochem J 2006 395 1 183 190 10.1042/BJ20051455 16390332 PMC1409686

[B19] Vives D Farage S Motta R Lopes AG Caruso-Neves C Atrial natriuretic peptides and urodilatin modulate proximal tubule Na(+)-ATPase activity through activation of the NPR-A/cGMP/PKG pathway Peptides 2010 31 5 903 908 10.1016/j.peptides.2010.02.018 20206222

[B20] Landgraf SS Wengert M Silva JS Zapata-Sudo G Sudo RT Takiya CM Pinheiro AA Caruso-Neves C Changes in angiotensin receptors expression play a pivotal role in the renal damage observed in spontaneously hypertensive rats Am J Physiol Renal Physiol 2011 300 2 F499 510 10.1152/ajprenal.00384.2010 21084406

[B21] Queiroz-Madeira EP Lara LS Wengert M Landgraf SS Libano-Soares JD Zapata-Sudo G Takiya CM Gomes-Quintana E Lopes AG Na(+)-ATPase in spontaneous hypertensive rats: possible AT(1) receptor target in the development of hypertension. Biochim Biophys Acta, 2010 1798 3 360 366 10.1016/j.bbamem.2009.06.018 19560439

[B22] Chiolero A Maillard M Nussberger J Brunner HR Burnier M Proximal sodium reabsorption: An independent determinant of blood pressure response to salt Hypertension 2000 36 4 631 637 10.1161/01.HYP.36.4.631 11040249

[B23] Biollaz J Waeber B Diezi J Burnier M Brunner HR Lithium infusion to study sodium handling in unanesthetized hypertensive rats Hypertension 1986 8 2 117 121 10.1161/01.HYP.8.2.117 3510975

[B24] de Souza AM Lara Lda S, Previato JO, Lopes AG, Caruso-Neves C, da Silva BP, Parente JP: Modulation of sodium pumps by steroidal saponins Z Naturforsch C 2004 59 5–6 432 436 18998415 10.1515/znc-2004-5-626

[B25] Kreydiyyeh SI Usta J Diuretic effect and mechanism of action ofPetroselinum hortense J Ethnopharmacol 2002 79 3 353 357 10.1016/S0378-8741(01)00408-1 11849841

[B26] Grubmeyer C Penefsky HS The presence of two hydrolytic sites on beef heart mitochondrial adenosine triphosphatase J Biol Chem 1981 256 8 3718 3727 6452454

[B27] Caruso-Neves C Coelho-Souza SA Vives D Goes G Lara LS Lopes AG Modulation of ouabain-insensitive Na(+)-ATPase activity in the renal proximal tubule by Mg(2+), MgATP and furosemide Int J Biochem Cell Biol 2002 34 12 1586 1593 10.1016/S1357-2725(02)00059-6 12379280

[B28] Marin R Gomez DC Rodriguez GA Proverbio T Proverbio F Ouabain-insensitive, Na-ATPase activity in pure suspensions of rat kidney proximal tubules FEBS Lett 1990 269 1 77 78 10.1016/0014-5793(90)81122-5 2167245

[B29] Gutkowska J Thibault G Januszewicz P Cantin M Genest J Direct radioimmunoassay of atrial natriuretic factor Biochem Biophys Res Commun 1984 122 2 593 601 10.1016/S0006-291X(84)80074-1 6235810

[B30] Bradford MM A rapid and sensitive method for the quantitation of microgram quantities of protein utilizing the principle of protein-dye binding Anal Biochem 1976 72 248 254 10.1016/0003-2697(76)90527-3 942051

[B31] Schulz-Knappe P Forssmann K Herbst F Hock D Pipkorn R Forssmann WG Isolation and structural analysis of "urodilatin", a new peptide of the cardiodilatin-(ANP)-family, extracted from human urine Klin Wochenschr 1988 66 17 752 759 10.1007/BF01726570 2972874

[B32] Gagelmann M Hock D Forssmann WG Urodilatin (CDD/ANP-95-126) is not biologically inactivated by a peptidase from dog kidney cortex membranes in contrast to atrial natriuretic peptide/cardiodilatin (alpha-hANP/CDD-99-126) FEBS Lett 1988 233 2 249 254 10.1016/0014-5793(88)80436-8 2968281

[B33] Abassi ZA Tate J Hunsberger S Klein H Trachewsky D Keiser HR Pharmacokinetics of ANF and urodilatin during cANF receptor blockade and neutral endopeptidase inhibition Am J Physiol 1992 263 5 Pt 1 E870 876 1443119 10.1152/ajpendo.1992.263.5.E870

[B34] Becker BN Greene J Evanson J Chidsey G Stone WJ Ginseng-induced diuretic resistance JAMA 1996 276 8 606 607 8773630 10.1001/jama.1996.03540080028021

[B35] Bangham AD Horne RW Glauert AM Dingle JT Lucy JA Action of saponin on biological cell membranes Nature 1962 196 952 955 10.1038/196952a0 13966357

[B36] Foussard-Guilbert F Ermias A Laget P Tanguy G Girault M Jallet P Detergent effects of kinetic properties of (Na++ K+)-ATPase from kidney membranes Biochim Biophys Acta 1982 692 296 304 10.1016/0005-2736(82)90534-X 6293563

[B37] Caruso-Neves C Vives D Dantas C Albino CM Fonseca LM Lara LS Iso M Lopes AG Ouabain-insensitive Na + −ATPase of proximal tubules is an effector for urodilatin and atrial natriuretic peptide Biochim Biophys Acta 2004 1660 1–2 93 98 14757224 10.1016/j.bbamem.2003.11.002

[B38] Citarella MR Choi MR Gironacci MM Medici C Correa AH Fernandez BE Urodilatin and dopamine: a new interaction in the kidney Regul Pept 2009 153 1–3 19 24 19101594 10.1016/j.regpep.2008.11.009

